# Orthopœdic Treatment of Paralysis of the Ocular Muscles

**Published:** 1878-01

**Authors:** 


Orthopædic Treatment of Paralysis of the Ocular Muscles.—The author, Prof. Michel, calls attention to a new method for the treatment of paralysis of the ocular muscles, based on the principle of *passive movements.*

The following is the method : the conjunctiva is grasped near the scelero-corneal border over the insertion of the paralyzed muscle, with the ordinary fixation forceps, and the globe of the eye turned in the direction of the paralyzed muscle, beyond the point it is usually carried by the normal contraction of the muscle, then back again, these motions back and forth to be continued about two minutes. He says the effects are greatest immediately after the operation and are quite astonishing. The improvement amounts to at least 1½ millimeters.

The pain of the process is slight and the conjunctival irritation readily subdued by cold compresses. One exercise a day is employed.

He claims as the chief advantages of the method: (1) elimination of the action of the antagonist muscles, (2) shorter duration of the treatment.—*The Clinic.*

